# Chemical Composition, Biological Activity, and In VivoToxicity of Essential Oils Extracted from Mixtures of Plants and Spices

**DOI:** 10.3390/molecules30234579

**Published:** 2025-11-28

**Authors:** Fouad Bahri, Antoni Szumny, Adam Figiel, Youcef Bahri, Aleksandra Włoch, Barbara Bażanów, Aleksandra Chwirot, Tomasz Gębarowski, Paulina Bugno, El Mokhtar Bahri, Rabia Nouria Benabdeloued

**Affiliations:** 1 Laboratory of Microbiology and Plant Biology, Faculty of Nature and Life Sciences, Abdelhamid Ibn Badis University, BP 188/227, Mostaganem 27000, Algeria; youcefbahri99@gmail.com (Y.B.); mokhtar.bahri.91@gmail.com (E.M.B.); benabdelouedrabia@gmail.com (R.N.B.); 2 Department of Food Chemistry and Biocatalysis, Wrocław University of Environmental and Life Sciences, Norwida 31, 50-375 Wroclaw, Poland; antoni.szumny@upwr.edu.pl; 3 Institute of Agricultural Engineering, Faculty of Life Sciences and Technology, Wrocław University of Environmental and Life Sciences, Chelmonskiego 37a, 51-630 Wroclaw, Poland; 4 Department of Physics and Biophysics, Faculty of Biotechnology and Food Sciences, Wrocław University of Environmental and Life Sciences, C.K. Norwida 25, 50-375 Wroclaw, Poland; aleksandra.wloch@upwr.edu.pl; 5 Department of Pathology, Wrocław University of Environmental and Life Sciences, Norwida 31, 50-375 Wroclaw, Poland; barbara.bazanow@upwr.edu.pl (B.B.); aleksandra.chwirot@upwr.edu.pl (A.C.); 6 Department of Biostructure and Animal Physiology, Faculty of Veterinary Medicine, Wrocław University of Environmental and Life Sciences, Norwida 31, 50-375 Wroclaw, Poland; tomasz.gebarowski@upwr.edu.pl (T.G.); paulina.bugno@upwr.edu.pl (P.B.)

**Keywords:** herbal and spice blends, essential oils, antimicrobial activity, antioxidant activity, chemopreventive activity, acute toxicity

## Abstract

The study focused on essential oils (EOs) of plant origin, which are of great interest to scientists in the context of medical applications due to their biological properties, such as antimicrobial, anti-inflammatory, antioxidant, and anticancer effects. The objective of the study was to determine chemical profiles and biological activities of the essential oils extracted from five mixtures (M1 [*Thymus vulgaris*, *Ammi visnaga*, *Syzygium aromaticum*, *Citrus sinensis*]; M2 [*Thymus vulgaris*, *Ammi visnaga*, *Cinnamomum verum*, *Citrus sinensis*]; M3 [*Mentha pulegium*, *Lavandula angustifolia*, *Zingiber officinale*, *Citrus sinensis*]; M4 [*Mentha pulegium*, *Lavandula angustifolia*, *Cinnamomum verum*, *Citrus sinensis*]; M5 [*Ammi visnaga*, *Lavandula angustifolia*, *Zingiber officinale*, *Syzygium aromaticum*]). Each mixture was derived from a blend of four selected plants used in traditional medicine in Mostaganem, Algeria. When selecting the best composition, the interactions between plant components were considered in terms of potential therapeutic benefits. The chemical compositions of the EO mixtures were analyzed using GC-MS. The acute toxicity of the EO mixtures was evaluated in vivo following oral administration. The sensitivity of the microorganisms to the EO mixtures was determined using the agar diffusion method. Virucidal testing was performed using the quantitative suspension method to determine virucidal activity, as described in the European standard for disinfectants used in the medical field. The antioxidant activity of the EO mixtures was evaluated using a model membrane system based on liposomes derived from soybean phosphatidylcholine. Chemopreventive activity was assessed in vitro using cell culture. The main compounds identified were carvacrol and thymol in M1; geranial, cinnamylaldehyde, and carvacrol in M2; pulegone and limonene in M3; geranial and cinnamylaldehyde and limonene in M4; and eugenol and caryophyllene in M5. The selection of the “best” blend depended on the biological activity deemed most critical for the specific application. Specifically, M3, M4, and M5 exhibited the strongest anti-HSV-1, anti-HAdV-5, and anticancer activity, respectively. In contrast, M1, a potent antioxidant, demonstrated the strongest antibacterial and anticancer activity. These results indicate that M1, M3, M4, and M5 EOs have promising applications in the pharmaceutical industry and medical research.

## 1. Introduction

Natural products are increasingly being studied because they are compatible with healthy tissues, making them attractive candidates for therapeutic use. This area of research is being further explored because of the benefits of phytochemicals, which can support immune function, neutralize free radicals, and contribute to cancer prevention [1,2].

Human adenovirus and herpes simplex virus are common viruses, but rapid transmission of their infections and the emergence of treatment-resistant strains pose major challenges to the immune system of the host. These viruses can affect many body systems (ocular, respiratory, digestive, urogenital, and central nervous system) and cause infectious diseases that can be particularly serious in vulnerable individuals, such as immunocompromised individuals, young children, and the elderly [3,4]. The development of new antiviral drugs and virucidal substances is crucial and requires exploration of alternative therapeutic approaches. The medicinal plants in the five herbal teas from our study, recognized for the diversity of their bioactive compounds and their still largely untapped potential, are increasingly considered promising sources of new antiviral agents [5,6].

The overuse and misuse of antibiotics, often driven by patient demand, has contributed to the alarming increase in multidrug-resistant (MDR) bacteria. Despite this growing threat, the development of new antibiotics remains limited, posing a major challenge to modern medicine. This escalating crisis underscores the urgent need to develop alternative therapeutic strategies to combat bacterial resistance [7]. The World Health Organization (WHO) has emphasized that this growing threat to public health is no longer a future concern but an urgent reality affecting all regions of the world [8]. To address this critical challenge, the WHO implemented a Strategy for Traditional Medicine for the period 2014–2026 [9].

Oxidative stress is an imbalance between pro-oxidant and antioxidant systems in the body. Excess free radicals are involved in the development of more than one hundred different human diseases [10,11]. Synthetic antioxidants, such as butylhydroxyanisole (BHA), butylhydroxytoluene (BHT), tert-butylhydroquinone (TBHQ), and propyl gallate (PG), raise safety concerns due to their potential toxicity. Recent studies suggest that they may contribute to liver damage and even carcinogenesis [12,13,14]. Medicinal plants are increasingly recognized as promising sources of novel antioxidant agents [15]. Furthermore, in recent decades, cancer has become a major global problem and the leading cause of death, and its incidence has increased over the years [16]. Synthetic drugs often represent the only therapeutic option for cancer treatment [17]. However, most of these agents destroy not only tumor cells but also healthy cells [18]. Therefore, there is an urgent need for new treatments with fewer side effects. The use of medicinal plants, and more specifically their secondary metabolites and essential oils (EOs), could be a promising alternative [19,20].

Faced with the various problems associated with the use of synthetic agents, the appeal of alternative natural sources has become increasingly important. Among these innovations is the use of essential oils (EOs) derived from blends of plants and spices, which offer enhanced biological activity thanks to their synergistic and additive effects [21,22].

The use of medicinal plant mixtures is an ancient practice. The inhabitants of Mostaganem (Algeria) inherited the tradition of preparing five herbal teas based on medicinal plants from the Islamic civilization of Andalusia to prevent respiratory, urinary, and digestive infections [23,24,25]. The preparation and use of these herbal teas is nowadays Performed in the same way as in Andalusian times. Each herbal tea was prepared by infusing four plants and spices. These herbal teas are used in the fall to prevent and treat microbial infections. This ancestral knowledge, passed down from generation to generation, testifies to the importance of traditional medicine for subsistence and health.

Each herbal tea is a mixture of four medicinal plants selected from the following species: *Thymus vulgaris* L., *Ammi visnaga* L., *Mentha pulegium* L., *Lavandula angustifolia* L., *Zingiber officinale* L., *Cinnamomum verum* L., *Syzygium aromaticum* L., and *Citrus sinensis* L.

The composition of these five herbal teas is as follows: herbal Tea 1 (*Thymus vulgaris*, *A. visnaga*, *S. aromaticum*, *C. sinensis*), herbal Tea 2 (*T. vulgaris*, *A. visnaga*, *C. verum*, *C. sinensis*), herbal Tea 3 (*M. pulegium*, *L. angustifolia*, *Z. officinale*, *C. sinensis*), herbal tea 4 (*M. pulegium*, *L. angustifolia*, *C. verum*, *C. sinensis*), herbal tea 5 (*A. visnaga*, *L. angustifolia*, *Z. officinale*, *S. aromaticum*).

Several studies have demonstrated the biological activity of the medicinal plants used to prepare the above-mentioned teas. *Thymus vulgaris* has antimicrobial and antioxidant properties [26]. *A. visnaga* has antioxidant, anti-inflammatory, diuretic, antispasmodic, and antibacterial properties [27]. *M. pulegium* has insecticidal, antioxidant, and antimicrobial properties [28]. *L. angustifolia* has numerous biological properties [29]. *Z. officinale* is an analgesic, anti-inflammatory, anticancer, antidiabetic, hepatoprotective, nephron-protective, and antioxidant agent [30]. *Cinnamomum verum* has antiviral and antimicrobial properties [31]. *S. aromaticum* has antibacterial, anticancer, antioxidant, antiviral, antithrombotic, and antiparasitic properties [32]. *Citrus sinensis* has anticancer, antimicrobial, antioxidant, and anti-inflammatory properties [33].

Despite the use of these plants in the daily diet (e.g., in the form of infusions), their biological properties and toxicity to normal and cancer cells have not been tested. It remains to be determined whether these plants can act synergistically. Such tests are important because of the complexity of the composition of the mixtures.

The essential oils of the plants and spices mentioned above are complex mixtures of volatile chemical compounds with recognized medicinal benefits. They contain a wide variety of substances, including odorous molecules such as terpenes (monoterpenes and sesquiterpenes) and oxygenated compounds such as alcohols, aldehydes, ketones, and esters, which are the source of their therapeutic properties and volatile nature.

Therefore, our study aimed to evaluate the antibacterial, virucidal, antioxidant, and anticancer effects of five herbal teas (M1–M5), each derived from a combination of four plants and spicesrich in EOs, used in traditional Mostaganem medicine. We sought to explore the interactions between these oils and to discover their possible synergistic or additive effects. Furthermore, their in vivo effects in mice were examined using pharmacological tests.

## 2. Results

### 2.1. Chemical Analysis of EOs

#### 2.1.1. Chemical Composition of EOs

The chemical compositions of the EOs from five mixtures prepared for testing, analyzed by GC-MS, are detailed in Table S1 and Figures S1–S5 (Supplementary Materials). Table 1 lists the compounds significantly present in the essential oils of the mixtures with a threshold of 1% (or higher).

The results presented in Table S1 indicate that the essential oils (EOs) of mixtures M1, M2, M3, M4, and M5 contained 56 volatile compounds, representing 99.779%, 100%, 100%, 99.999%, and 99.924% of the total composition, respectively. On the other hand, the analysis of the content of the main volatile compounds listed in Table 1 shows that the main components of each mixture are as follows: M1 consists of carvacrol (41.098%), thymol (25.095%), and limonene (6.313%); M2 contains geranial + cinnamylaldehyde (32.146%), carvacrol (21.933%), thymol (15.954%), and limonene (10.438%); M3 is composed of pulegone (24.542%), limonene (22.64%), eugenol (15.556%), and zingiberene (5.298%); M4 includes geranial + cinnamylaldehyde (50.675%), limonene (12.403%), carvacrol (8.891%), and thymol (6.26%); and M5 consists of eugenol (61.042%), caryophyllene (10.272%), and thymol (8.553%).

#### 2.1.2. Chemical Class Composition of EOs

**Figure 1:**
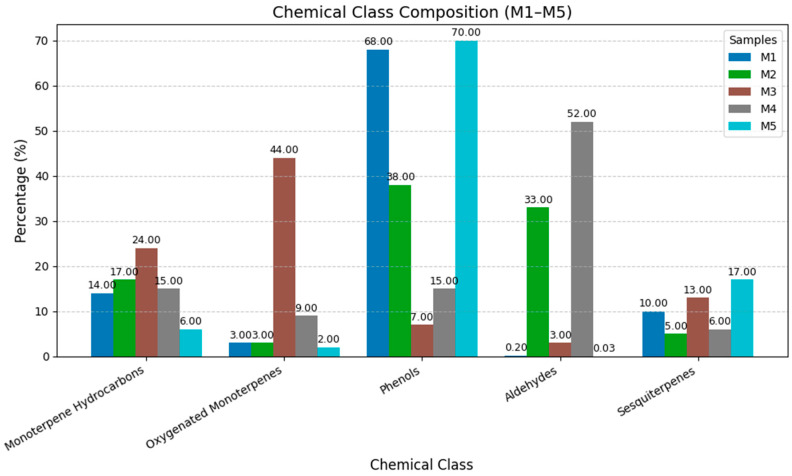
Comparative analysis of chemical class composition across.

Analysis of the chemical class composition revealed significant variations among the five essential oil samples (Figure 1). M1 was predominantly composed of phenols (68.0%), with moderate amounts of monoterpene hydrocarbons (14.0%) and sesquiterpenes (10.0%). This suggests potential antioxidant and antimicrobial properties. M2 exhibited a more balanced composition, with significant amounts of phenols (38.0%) and aldehydes (33.0%). The presence of aldehydes may contribute to distinctive aromatic properties. M3 was characterized by a high concentration of oxygenated monoterpenes (44.0%), which often contribute to the fragrance and therapeutic properties of essential oils. It also contains the highest proportion of monoterpene hydrocarbons (24.0%). M4 had the highest aldehyde content (52.0%) among all samples, suggesting strong aromatic characteristics. The composition is generally relatively balanced across other chemical classes. M5 showed the highest phenol content (70.0%) and a substantial sesquiterpene content (17.0%), indicating potential strong biological activity and stability.

### 2.2. Antimicrobial Activity of EOs

The disk diffusion method was used to determine the antibacterial activity of the oils tested against six multidrug-resistant bacteria [34]. The results were compared with those of standard antibiotics (positive control). The results are shown in Table 2.

The EOs of the five mixtures exhibited highly significant antibacterial effects against the six tested multidrug-resistant bacteria. The diameters of the halo ranged from 17.45 to 40.23 mm for M1, from 16.18 to 35.12 mm for M2, from 11.32 to 38.32 mm for M3, from 15.25 to 39.36 mm for M4, and from 14.14 to 39.08 mm for M5. The five mixtures showed significant antibacterial activity compared to standard drugs (Table 2).

The EOs of the mixtures exhibited high activity against the six strains tested (Table 3). The values were obtained in a range from 0.312 to 10 μL/mL for the MIC and from 0.312 to C 10 μL/mL for the MBC. The susceptibility was particularly high in *E. coli* toward M1, M3, and M5, *P. aeruginosa* toward M2 and M5, *E. Hormaechi* toward M5, and *S. aureus* toward M1 and M4. The MIC and MBC were both recorded at the lowest value, which was approximately 0.312 μL/mL. On the other hand, the sensitivity is much lower in *P. aeruginosa*; the MIC and MBC are obtained at higher values, from 1.25 to 10 μL/mL for M3.

Based on the MIC and MBC results of the EOs mixtures, we observed that the EOs mixtures were bactericidal against the bacteria tested, except for M3 which was bacteriostatic against *P. aeruginosa* (Table 3). An MBC/MIC ratio of an antibacterial substance less than or equal to 4 can be considered bactericidal, but if the ratio is greater than 4, it is bacteriostatic [35].

### 2.3. Virucidal Activity

**Figure 2:**
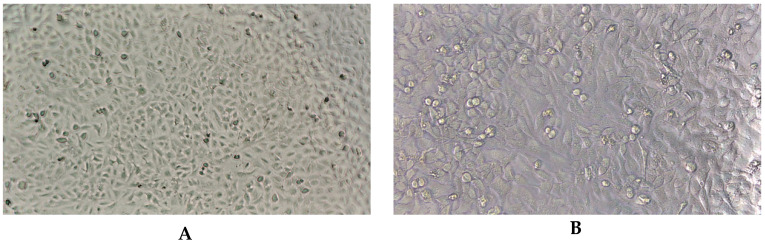

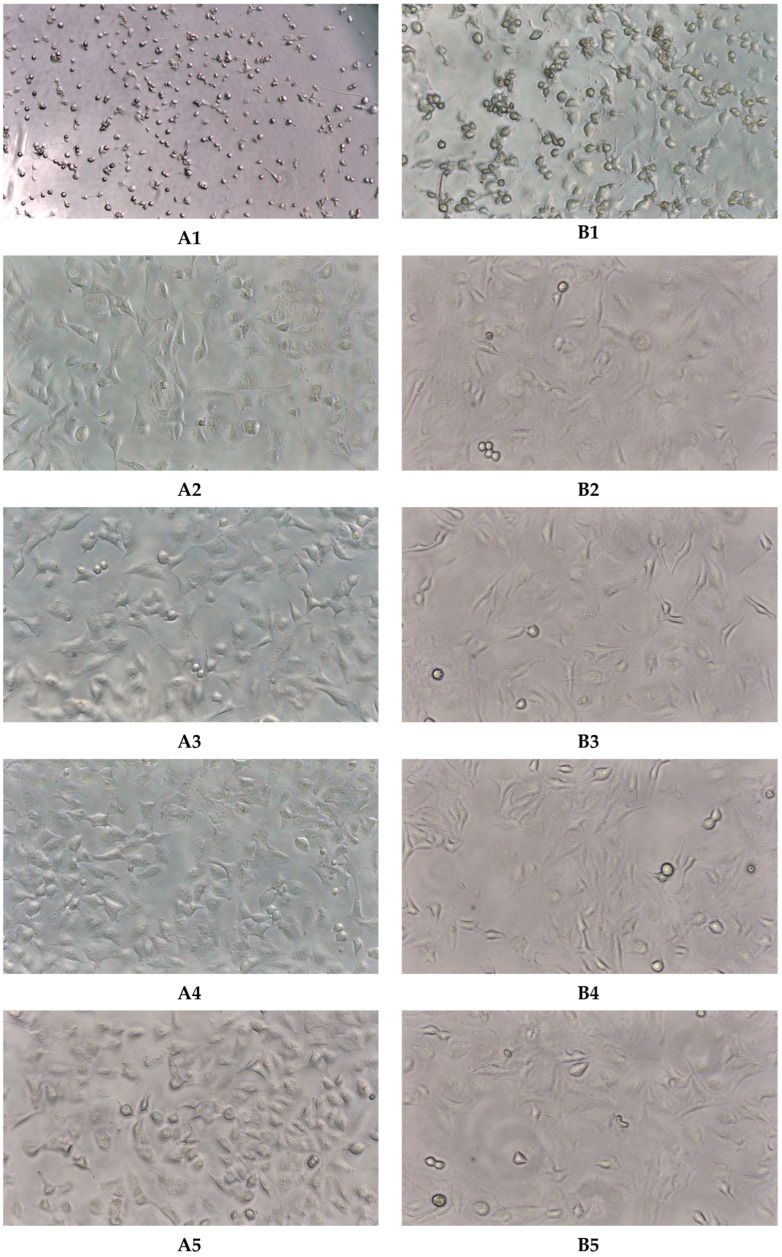

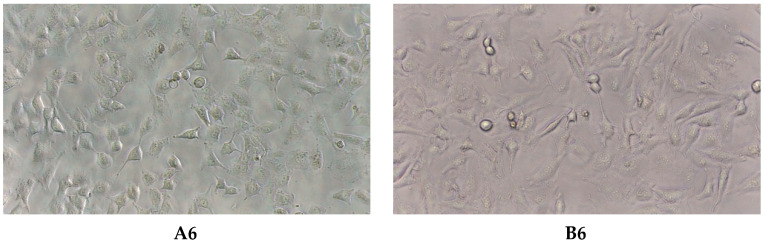
The antiviral potential of essential oils against human adenovirus type 5 and human herpes simplex type 1 was assessed using a method established by the European Committee for Standardization (Brussels, Belgium, 2013) that evaluates the efficacy of products in inactivating viruses. (**A**,**B**)—negative control, noninfected A549 (**A**) and HeLa (**B**) cell lines; (**A1**,**B1**)—positive control, cytopathic effect caused by HAdV-C5 (**A1**) and HSV-1 (**B1**); (**A2**–**A6**)—A549 cells inoculated with the HAdV-C5 virus and HAdV-C5 with essential oils M1, M2, M3, M4, M5 essential oils, respectively; (**B2**–**B6**)—HeLa cells inoculated with HSV-1 virus and HSV-1 with M1, M2, M3, M4, M5 essential oils, respectively.

The essential oils of the blends were evaluated for their potential to inactivate viruses, particularly herpes simplex virus type 1 (HSV-1) and human adenovirus (HAdV-5), as listed in Table 4. The essential oils of the blends demonstrated a significant reduction in viral titers, reaching a reduction rate of ≥4 log (99.99%) for HSV-1 and HAdV-5. The photographs (Figure 2) illustrate the absence of CPE due to the inhibition of viral replication by the essential oils of the tested blends.

### 2.4. Antioxidant Activity

**Figure 3:**
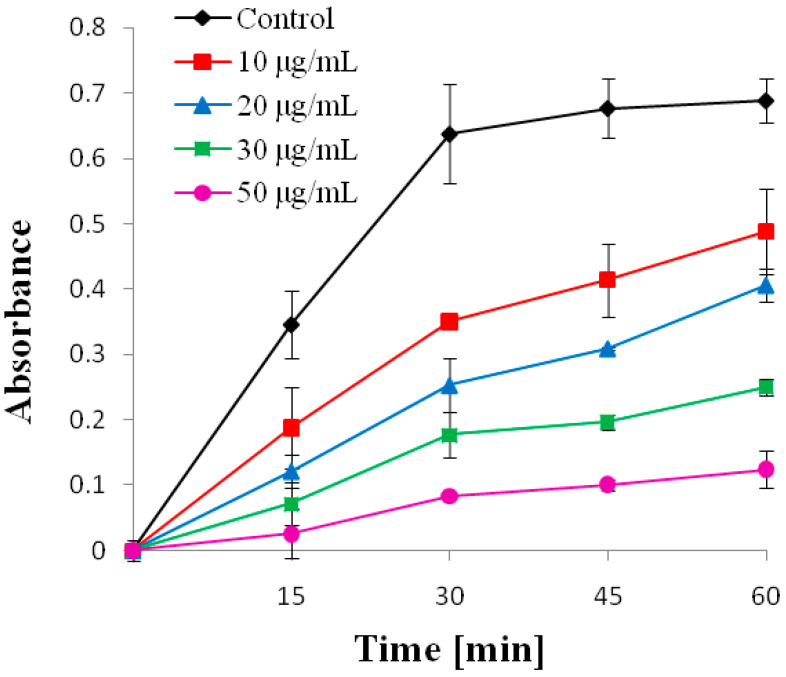
The kinetics of model membrane oxidation caused by UVC radiation for 60 min in the presence of different concentrations of M5. The absorbance data represent the absorbance values after background correction.

To determine the effect of the essential oils from the five mixtures on the kinetics of lipid oxidation in the model membrane, concentration-dependent absorption curves were prepared after 1 h of irradiation. Figure 3 shows an example of the oxidation curve for the M5 mixture.

As shown in the figure, the absorption increased with increasing UV exposure time, indicating an increase in membrane lipid oxidation. Simultaneously, it can also be observed that as the concentration of the essential oils increased, absorption decreased compared to the control, indicating that the compound inhibited the lipid oxidation process. For the highest irradiation time (1 h), the percentage inhibition of oxidation of individual mixtures was determined, as well as the concentration responsible for 50% inhibition of lipid oxidation (IC_50_) was determined. This parameter indicates that the essential oils, composed of different plants, have different antioxidant activities. Table 5 shows the IC_50_ values for the individual mixtures. The results showed that the lowest IC_50_ value of 14.02 ± 3.72 μg/mL was found for mixture M1, and the highest for M3, with an IC_50_ of 281.60 ± 23.78 μg/mL. (Table 5) The IC_50_ of the M2 and M5 were similar, at 27.75 ± 3.41 and 25.90 ± 0.34 μg/mL, respectively. The obtained values were compared with those of standard antioxidants such as Trolox^®^ and ascorbic acid (AA). The IC_50_ values obtained using the same method were 10.0 ± 1.46 μg/mL for Trolox^®^ [36] and 16.6 ± 2.6 μg/mL for AA [37]. The low IC_50_ value obtained for M1, comparable to that of standard antioxidants, indicates a very high antioxidant activity of this mixture. In contrast, the very high IC_50_ value of mixture M3 indicates a very low antioxidant activity. Accordingly, the antioxidant activity decreased in the following order: M1 > M5 > M2 > M4 > M3. The composition of the individual plants and the content of the main components were likely responsible for the antioxidant activities of the mixtures. It can be seen that in all mixtures, except M3, thymol, carvacrol, and eugenol were the main components (Table 1), and their higher content corresponded to the higher activity of the individual mixtures.

### 2.5. Chemopreventive Activity

**Figure 4:**
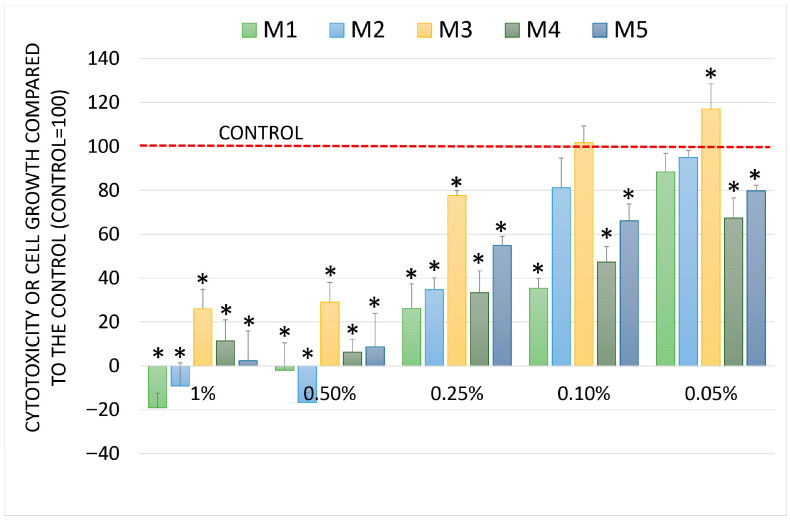
Cell growth and cytotoxicity (SRB assay) after incubation with test oils at different concentrations in human NHDF fibroblasts. Data are presented as mean ± standard deviation (SD). Sign * denotes a significant difference compared to the negative control for all oils and concentrations at *p* < 0.05.

The chemopreventive activity was evaluated in four human cell lines: fibroblasts and three tumor lines. The Sulforhodamine B (SRB) assay was used to evaluate biological activity, which measures cell growth and cytotoxicity depending on the effect of the compound under study. Cell growth was relative to that of the control (100). When the results were above 100, it indicated growth stimulation, ranging from 0 (control T0–start of the test) to 100 (control) cytostatic effect, and when the results were below 0, it indicated a cytotoxic effect. The results for the normal cells are shown in Figure 4. The tested oils inhibited cell growth. In contrast, the cytotoxic effect was the strongest for a mixture of oils at a concentration of 1%. The inhibition of cell growth in fibroblast cultures was concentration-dependent and gradually decreased up to a concentration of 0.1%. The lowest cell toxicity was observed for oils M2 and M3.

**Figure 5:**
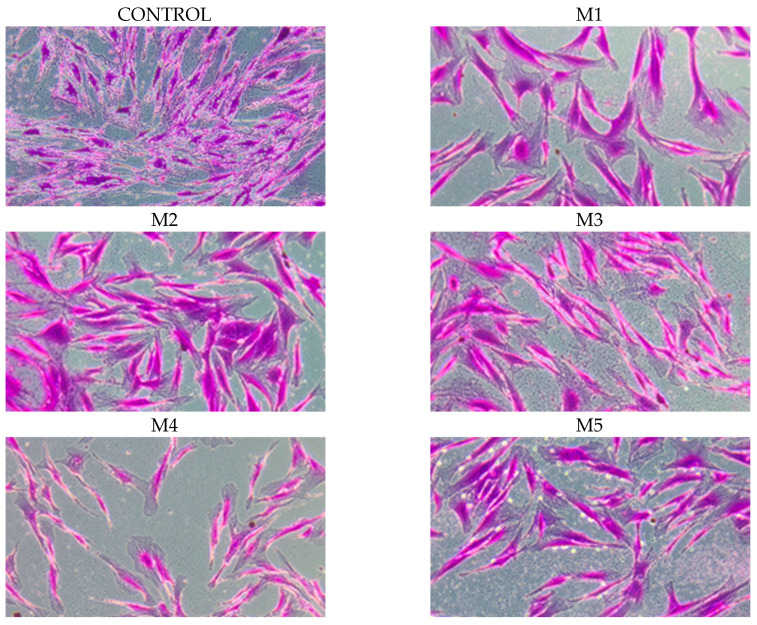
The essential oils in the mixtures were tested on four adherent cell lines in vitro. NHDF were fixed and stained with sulforhodamine B at a 10× objective magnification. The microphotographs show the following in order: control–cells not incubated with essential oils, cells incubated with essential oils M1–M5 at a concentration of 0.1%.

The results obtained from the SRB test performed on human NHDF fibroblasts were confirmed using photographs (Figure 5).

**Figure 6:**
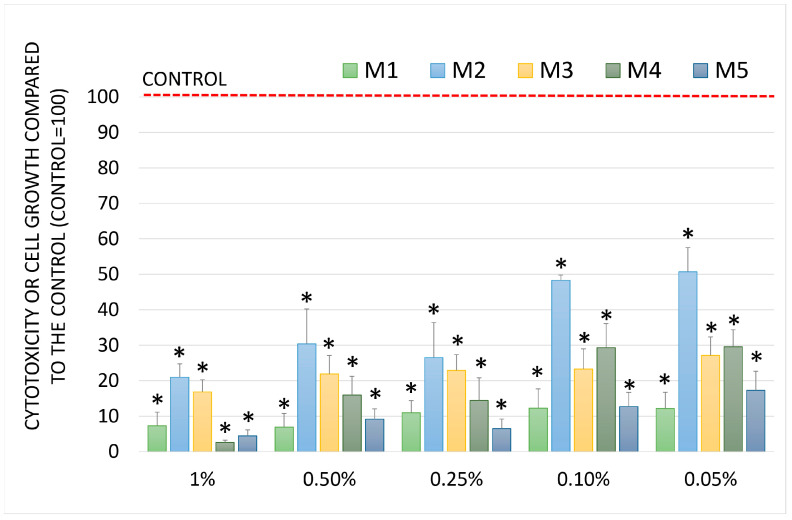
Cell growth and cytotoxicity (SRB assay) after incubation with essential oils at different concentrations in A549 lung adenocarcinoma cells. Data are presented as mean and SD. Sign * denotes a significant difference compared to the negative control for all oils and concentrations at *p* < 0.05.

Another line in which the tests were performed was A549 lung adenocarcinoma. The results are shown in Figure 6. This line was chosen because the essential oils are often used for aromatherapy; therefore, their anticancer properties in reducing the risk of lung cancer can be very interesting. In this study, only inhibition of cell growth was observed.

Essential oils M1 and M5 inhibited the growth of the tested cells the most. Essential oil M2 showed the weakest growth inhibition. The cell growth inhibitory activity of all essential oils across the entire concentration range was concentration-dependent and statistically significant.

**Figure 7:**
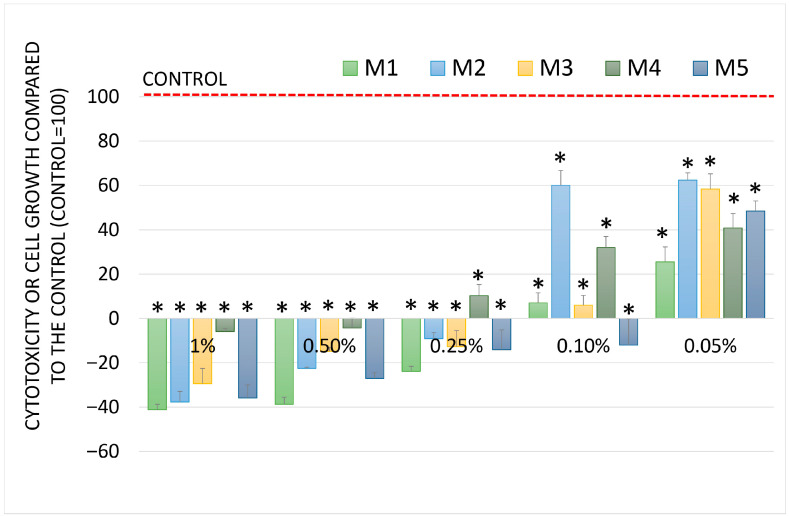
Cell growth and cytotoxicity (SRB assay) after incubation with the essential oils at different concentrations in LoVo colon adenocarcinoma cells. Data are presented as mean and SD. Sign * denotes a significant difference compared to the negative control for all oils and concentrations at *p* < 0.05.

Colorectal cancer (CRC) is a major clinical problem and a disease of civilisation. The search for new substances with activity against this type of cancer is an important research problem. Inhaled essential oils can also enter the gastrointestinal tract and affect the altered cells therein. Some also use essential oils as food additives, that is, an additive to flavored cakes, teas, and coffee. The LoVo colorectal cancer cell line was selected for this study. It is commonly used in studies to assess chemopreventive activities. In contrast, essential oils in the concentration range of 0.25 to 1% showed strong cytotoxicity, while at concentrations of 0.1 and 0.05%, they inhibited cell growth. M1 and M5 essential oils demonstrated the strongest cytotoxic activity against colorectal cancer cells, similar to that of lung cancer cells. The weakest values were for M2 and M4. The results were concentration-dependent and statistically significant. The results are presented in Figure 7.

**Figure 8:**
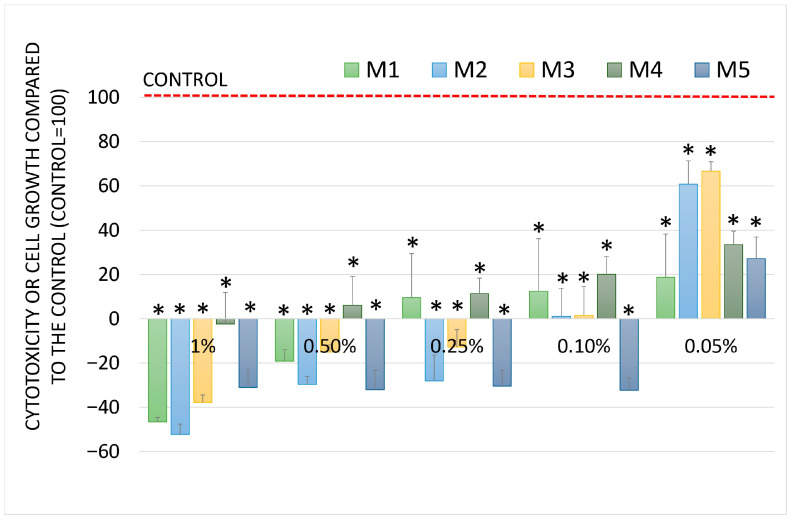
Cell growth and cytotoxicity (SRB assay) after incubation with essential oils of the test at different concentrations in MCF7 breast adenocarcinoma cells. Data are presented as mean and SD. Sign * denotes a significant difference compared to the negative control for all oils and concentrations at *p* < 0.05.

The last cell line tested was MCF-7, a hormone-dependent breast cancer cell line. The results of this study are shown in Figure 8. The aim of this study was to conduct a preliminary assessment of the cytotoxic and chemopreventive potential of selected essential oil compositions, as well as to determine their possible use as adjuvants in cancer therapy. In this cell line, a cytotoxic effect was observed in a series from 0.1 to 1%. The essential oil M5 had the strongest effect on tumor cells, while M4 had the weakest effect. A cell growth-inhibiting effect was observed even at the lowest essential oil concentration (0.05). The results demonstrated a significant biological effect at all concentrations and essential oils tested.

**Figure 9:**
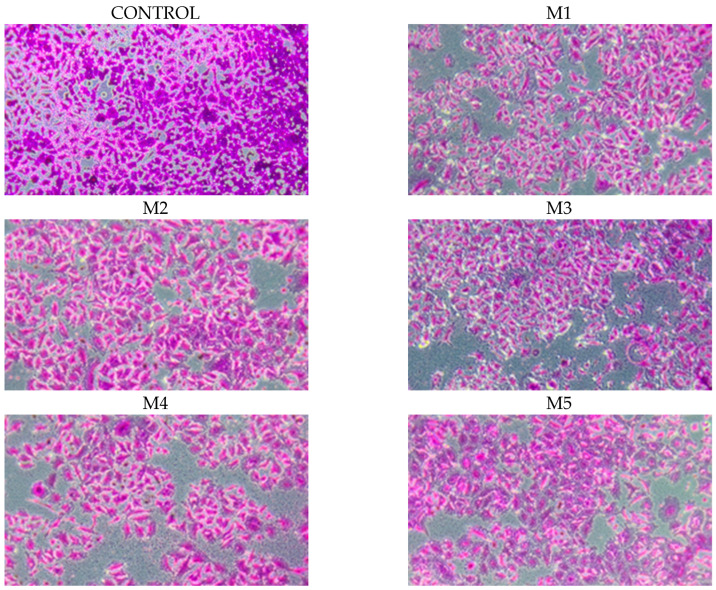
Essential oils in the mixtures were tested on four adherent cell lines in in vitro cell culture.Tumor cells MCF-7 were fixed and stained with sulfur red B at a 10× objective magnification. The microphotographs show, in order: control, cells not incubated with essential oils, and cells incubated with essential oils M1–M5, at a concentration of 0.1%.

The results obtained from the SRB test performed on MCF-7 tumor cells were confirmed using photographs (Figure 9).

### 2.6. Acute Oral Toxicity

The study of the acute toxicity of the mixtures revealed that no mortality was recorded in the tested mice, but sedation was observed during the first few hours after treatment at 5000 mg/kg.

## 3. Discussion

The exploration of natural substances by the scientific community is motivated by their potential to offer safer alternatives to synthetic products, particularly in medicine (drugs). One of these innovations is the use of essential oils (EOs) derived from mixtures of plants and spices, which offer enhanced biological activity due to their synergistic and additive effects. Our research aims to study the antibacterial, virucidal, antioxidant, and anticancer effects of five complex essential oils, obtained from combinations of four plants and spices from the traditional medicine of Mostaganem. This study also aimed to analyze the possible interactions between these oils to determine whether their effects were additive or synergistic.

The EOs yields obtained by hydrodistillation were 1.83%, 2.13%, 2.52%, 2.65%, and 3.2% for the M2, M4, M1, M3, and M5 blends, respectively, on a dry weight basis. These yields at 1% and above are generally considered good to excellent, as it indicates a higher oil concentration, which impacts the cost, quality, and sustainability of production [38].

The chemical profile of essential oil (EOs) derived from the plant mixture is the combination of the chemical profiles of EOs derived from individual plants that compose this mixture. *Thymus vulgaris* and *Ammi visnaga* are dominated by thymol and limonene, characteristic of thyme species, and provide a strong herbaceous and lemony base. *Syzygium aromaticum* contains exclusively eugenol. *Cinnamomum verum* is rich in eugenol, limonene, and caryophyllene. *Citrus sinensis* contains exclusively limonene. *Zingiber officinale* contains limonene and zingiberene. *Mentha pulegium* contains eugenol, thymol, and limonene. *Lavandula angustifolia* contains eugenol, limonene, and caryophyllene.

The main components identified in the EOs of the five mixtures are carvacrol, thymol, and limonene for M1; geranial, cinnamylaldehyde, carvacrol, thymol and limonene for M2; pulegone, limonene, eugenol, and zingiberene for M3; geranial, cinnamylaldehyde, limonene, carvacrol, and thymol for M4; and eugenol, caryophyllene, and thymol for M5. These components possess, according to the literature, a wide range of bioactivities potentially useful for clinical applications, such as antibacterial [39,40,41,42,43,44,45,46,47], antioxidant [47,48,49,50,51,52,53,54,55], antiviral [41,42,43,44,45,46,47,48,49,50,51,52,53,54,55,56,57,58,59,60,61,62,63,64], and anticancer activity [65,66,67,68,69,70,71,72,73].

The mixture M1 is characterized by the presence of phenolic monoterpenes and one monoterpene. Carvacrol and thymol are potent broad-spectrum antibacterial agents [40,41]. Limonene is known for its antiviral properties [57,61]. The M2 consists of geranial, cinnamaldehyde, carvacrol, thymol, and limonene. This mixture combines the properties of the components of M1 with those of the aldehyde and the citral isomer. Geranial and cinnamaldehyde are known for their antibacterial activity [43,45]. Carvacrol, thymol, and limonene complete the profile of this mixture. The M3 consists of pulegone, limonene, eugenol, and zingiberene. This mixture is more complex, including a potentially toxic ketone, terpene, phenol, and sesquiterpene. Pulegone, found in pennyroyal, is neurotoxic. It must be used with extreme caution. Limonene provides antiviral properties [44,46]. Eugenol is a phenol known for its antibacterial [45,47] and antiviral [61,62] properties. Zingiberene is a sesquiterpene found in the essential oil. Blend M4 is composed of geranial, cinnamaldehyde, limonene, carvacrol, and thymol. This blend is similar to M2 and combines the properties of aldehydes, phenols, and monoterpenes. Geranial and cinnamaldehyde are known for their strong antibacterial activity [42,43]. Limonene, carvacrol, and thymol are known for their antibacterial properties [44,46]. Blend M5 is composed of eugenol, caryophyllene, and thymol. This blend is distinguished by the presence of eugenol and caryophyllene, a sesquiterpene. Eugenol is an antibacterial [45,47] and antiviral [64] phenol. Caryophyllene is a sesquiterpene found in many essential oils, especially clove. Thymol is a phenol with potent antibacterial properties [45,74].

Traditional herbal remedies generally consist of a mixture of plants. We noticed that EOs from a mixture of plants are richer in compounds with biological effects than EOs from a single plant. Several studies have confirmed these results [75,76,77,78,79,80,81,82,83,84].

EOs of the five mixtures demonstrated bactericidal activity against most of the bacteria tested. These bacteria are multiresistant to antibiotics and are responsible for nosocomial infections. This bactericidal activity has been attributed, as mentioned in the literature, mainly to the individual components of the EOs in the mixtures: carvacrol [74,85,86,87,88], cinnamylaldehyde [81], and eugenol [45,89] thymol [45,88], limonene [74,90], geranial [91], caryophyllene [92], zingiberene [93], P-cymene [45], pulegone [94], γ-terpinene [95], β-bisabolene [96] α-curcumene [97], β-sesquiphellandrene [98], linalool [99], menthone [100], δ-Cadinene [101], endo-Borneol [102], copaene [103].

The antibacterial activity of EOs in the blends may also be due to the synergistic and additive effects of these organic compounds [104,105,106,107,108,109]. The synergistic effects of the minor constituents may also play a role [110]. Although the mechanisms associated with the antibacterial activities of EOs are not completely clear [111], the number of studies in this area is increasing [112]. The main organic compounds in the mixture make the cell membrane permeable [40,41,45,92,113,114,115,116,117,118,119,120], inhibit transcription, DNA translation, differential expression of various proteins and enzymes involved in transport, respiration, metabolism, chemotaxis, and protein synthesis [41,92,116,119,120,121], alter the structural components of the membrane and the corresponding properties [100], alter cell morphology and damage the cell membrane by increasing membrane permeability and affecting membrane potential [101], and increase cell membrane permeability by forming cell membrane pores, resulting in leakage of cellular contents [103]. The antibacterial activity of the five EOs on microorganisms is as follows: M1 > M4 = M5 > M2 > M3.

Herpes simplex virus type 1 (HSV-1) and human adenovirus (HAdV-5) infections are spreading worldwide [122]. Although therapies are available, their safety and efficacy are limited by adverse effects and drug resistance [123,124]. Therefore, novel natural antivirals have been used, such as EOs, which are natural products with promising biological activities to exert effects on potential pharmacological targets of viruses. The EOs from the five mixtures inhibited the replication of herpes simplex virus type 1 (HSV-1) and human adenovirus (HAdV-5). Mixture M3 was the most potent against HSV-1, whereas mixture M4 was the most potent against HAdV-5. M2, M4, and M5 show strong, balanced activity against both viruses. This inhibition could be mainly due to the main components of the EOs of the M1-M5 mixtures, namely: eugenol, thymol, limonene, geranial, cinnamylaldehyde, carvacrol, caryophyllene, zingiberene, p-cymene, pulegon, γ-terpinene, β-bisabolene, α-curcumin, β-sesquiphellandrene, and linseed oil. Several studies have demonstrated the antiviral activity of the main components of essential oils of M1-M5 mixtures [99,125,126,127,128,129,130,131,132] in HSV-1 and HAdV-5. The virucidal activity of the mixtures essential oils from the tested in HSV-1 and HAdV-5 could also be due to the synergistic and additive effects of the different major and minor organic compounds [133].

Several studies have described the inhibitory effects of EOs from plants used in the preparation of M1-M5 mixtures on the viral replication cycle of HSV-1 [125,134,135,136,137,138,139,140].

The main mechanism of virucidal action of EOs is capsid disintegration and viral expansion, thus preventing the virus from infecting host cells by adsorption via the capsid [141]. EOs also inhibit hemagglutinin, a viral membrane protein essential for host cell entry, facilitating viral binding to host cells and fusion of its membrane with the cellular endosome, thus allowing viral genetic material to enter and initiate infection [140,142]. EOs and their components can inhibit late viral processes by targeting the redox signaling pathway through their compounds such as phenols and terpenes, thereby weakening the pathogen and suppressing the later stages of the viral cycle [141,143]. EOs are lipophilic, that is, fat soluble, and this property allows them to easily penetrate viral lipid membranes, disrupt their structure, and ultimately cause the virus to disintegrate [144].

Studies have shown that essential oils exhibit antioxidant activity against model membranes formed from soy lecithin. However, this effect varied depending on the compound tested. Mixture M1 showed by far the strongest antioxidant activity, with an I_C50_ value almost twice as low as the next best blend (M5). Furthermore, M3 is the least effective antioxidant. The antioxidant activity of the mixtures was probably due to the composition of the individual plants and the content of the main components. It can be seen that in all mixtures, except M3, thymol, carvacrol, and eugenol were the main components (Table 1), and their higher content corresponded to the higher activity of the individual mixtures. Essential oils exert antioxidant effects by donating hydrogen atoms (hydrogen atom transfer) to free radicals, forming stable, less reactive radicals and disrupting oxidative chain reactions, a well-documented process for phenolic compounds like carvacrol and thymol found in many essential oils [133,145]. Other effects include the stimulation of antioxidant enzymes such as superoxide dismutase (SOD) and catalase, and the prevention of the formation of free radical-generating enzymes, thereby reducing overall cellular oxidative stress [146].

The in vitro toxicity analysis clearly indicates that the tested compounds exhibit selective activity toward cell lines, which is dependent on the cell type. The compounds showed significantly lower toxicity to normal human NHDF fibroblasts compared to cancerous cell lines. The compound M3 demonstrated the lowest toxicity to normal human NHDF fibroblasts. The toxicity of the compounds also varied depending on the cancer cell lines: MCF7, A549, and LoVo. The MCF7 breast cancer cell line was shown to be the most sensitive to the compounds tested, showing strong cytotoxicity at a concentration of as low as 0.10%. Among the essential oils tested, M5 had the strongest effect on tumor cells, while M4 had the weakest. The lung cancer cell line A549 was the least sensitive to the compounds. For this particular cell line, compound M2 showed the weakest inhibitory effect on cell growth, while M1 and M5 showed the strongest. Differences in sensitivity of the individual cell lines suggest that the effectiveness of these compounds may depend on the type of tumor. Compound M5 showed strong effects on all cancer lines, making it a promising candidate for broader applications.

This activity is due to the components of the tested essential oils, including carvacrol [65,147], thymol [66,147], limonene [67,148], geranial [68,81], cinnamylaldehyde [69,149], pulegone [70,150], and eugenol [71,151], zingiberene [152,153,154] and caryophyllene [11,73].

EOs fight cancer by inducing apoptosis (programmed cell death), interrupting the cell cycle, preventing DNA damage (antimutagenic), acting as antioxidants, stimulating detoxification processes, and sometimes exerting synergistic effects with chemotherapy. These effects are mediated by the different chemical components of EOs, such as phenols and terpenoids, which can damage cancer cell membranes, alter cellular pH, and disrupt mitochondrial function [155,156,157].

The M1 and M5 are the most potent against tumor cells. Mixture M3 shows the highest selectivity (lowest toxicity to normal cells), which could be advantageous for certain therapeutic windows.

The essential oils in the blends (M1 to M5) are carefully formulated to best utilize the active compounds from their original plants. These five herbal teas exhibit a subtle balance of phenolic compounds (thymol, carvacrol, eugenol) for their potency, terpenes (limonene, caryophyllene, zingiberene) for their volatility and complementary effects, and aldehydes (geranial, cinnamaldehyde) for enhanced bioactivity and richer aromatic complexity. These five herbal teas originate from the Islamic civilization of Andalusia (711–1492). They have been prepared to create blends with properties superior to those of their individual components.

The blends (M1 to M5) harness the chemical synergy between compounds from different families (phenols, aldehydes, terpenes) to enhance biological activity. On the contrary, the individual oils (LTv to LLa) retain the natural chemical signature of their original plants, making them ideal as base oils or reference profiles for formulation.

## 4. Materials and Methods

### 4.1. Plant Material

The leaves of *T. vulgaris* (LTv), *A. visnaga* (LAv), *M. pulegium* (LMp), *L. angustifolia* (LLa), and orange peel *C. sinensis* (PCs) were harvested in spring 2024 in different regions of western Algeria (Table 6). Roots *of Z. officinale* L. (RZo), Bark of *C. verum* L. (BCv), and Clove: *S. aromaticum* L. (CSa) were purchased commercially.

The plant samples were identified by Professor Benhassaini Hachemi of the University of Sidi Bel Abbès. Reference specimens (Nos. BVSTv-27, BVSAv-46, BVSLa-13, BVSMp-27 and BVSCs-22) were deposited in the Laboratory of Microbiology and Plant Biology at the University Abdelhamid Ben Badis University of Mostaganem, Algeria. The components of the five mixtures were washed, air-dried in the dark at room temperature for 15 days, finely ground, and thoroughly mixed in polyethylene food containers.

### 4.2. Preparation of Mixtures

The mixtures were prepared by manual weighing using traditional pharmacognosy methods. The 100 g mixtures used for extraction were composed of four plants or spices, as shown in Table 7. These mixtures were hydrodistilled at boiling point for 3 h using a Clevenger-type apparatus (Labo24.pl, Gliwice, Poland).

The hydrodistilled essential oils were stored at 4 °C until analysis.

The content of hydrodistilled essential oils in the M1–M5 mixtures weighing 100 g each, taking into account the amounts obtained from the individual plant and spice components of the mixtures according to the literature, is listed in Table 8 [45,70,71,74,83,85,88,93].

### 4.3. GC-MS Analyses

Isolation, identification, and quantification of compounds present in EOs were carried out using a Bruker SCION 436-GC-MS/MS (Billerica, MA, USA) equipped with an SH-5 MSi capillary column (30 m × 0.25 mm × 0.25 µm; Shimadzu, Kyoto, Japan). Ten mL of EOs dried with anhydrous sodium sulfate (VI) were dissolved in dichloromethane (LABIOCHEM, Wroclaw, Poland) and injected with a volume of 1 µL at 220 °C (split ratio 50, helium as quarry gas with a flow rate of 1 mL/s).

The GC oven temperature program was run at 50 °C for 2 min, then at 160 °C at a rate of 1 °C/min, then at 300 °C at a rate of 15 °C/min and held for 10 min. The total program time was 129.33 min. The operating conditions for the MS were as follows: ion source temperature, 250 °C; interference temperature, 250 °C, scan 35–400 *m*/*z*. The following methods were used to identify HE components: (1) comparison of mass spectra obtained with the NIST 20 (National Institute of Standards and Technology) and FFNSC (Mass Spectra of Flavours and Fragrances of Natural and Synthetic Compounds) databases; (2) comparison of linear retention indices (LRI) calculated using a retention index calculator with the values presented in NIST 23 and FFNSC; and (3) comparison of retention times of unknown compounds with authentic standards. Standards for the EOs compounds were purchased from Sigma-Aldrich, MetaSci (Toronto, ON, Canada), Extrasynthèse (Genay, France), and the Naturalyze group’s natural products collection (Wrocław University of Environmental and Life Sciences). Two software packages were used for analysis: AMDIS (v. 2.73) and Spectrus v. 1.2 Build 126765 March 2022 (Advanced Chemistry Development, Toronto, ON, Canada). For the calculation of retention indices, the macro was published by Lucero [158].

### 4.4. Antibacterial Activity of EOs

#### 4.4.1. Microbial Strains

The EOs of the five mixtures were tested against *Escherichia coli*, *Pseudomonas aeruginosa*, *Enterobacter hormaechei*, *Enterobacter auxiensis*, *Klebsiella pneumoniae*, and *Staphylococcus aureus*. Multi-resistant clinical bacteria were isolated, identified, and genetically characterized at the Chlef Hospital (Algeria) by Boussena [159]. They are responsible for nosocomial infections.

#### 4.4.2. Aromatogram Technique (Vincent Method)

The antibacterial activity of EOs was assessed using the disk diffusion method [34,160]. In brief, 100 μL of a bacterial suspension, prepared in sterile 0.9% NaCl solution and adjusted to 10^8^ CFU/mL, were spread onto Petri dishes containing Mueller-Hinton agar. Blank sterile antibiotic disks (6 mm in diameter) were impregnated with 15 μL of EOs and placed on the inoculated agar. disks without EOs served as negative controls, while positive controls included antibiotic disks of ertapenem (10 µg) and rifampicin (30 µg). The plates were then incubated at 37 °C for 24 h and allowed to stand at room temperature for 30 min. Antibacterial activity was evaluated by measuring the diameter (mm) of the inhibition zone in triplicate.

#### 4.4.3. Determination of Minimum Inhibitory Concentration (MIC)

The MIC values were determined using the broth macrodilution method. The EOs emulsion was serially diluted twice in a 0.2% agar solution [161]. The final concentrations ranging from 0.0781 to 10 μL/mL (*v*/*v*) were tested against the selected microbial strains. A volume of 0.2 mL from each EOs dilution was added to test tubes containing 1.8 mL of bacterial culture in Mueller Hinton broth, standardized to 5.10^5^–5.10^7^ bacteria/mL. The tubes were then incubated at 37 °C for 24 h [162]. A negative control, consisting of nutrient broth and inoculum without EOs, and positive controls with sterile broth and each EOs concentration, were included. Each EOs concentration was tested in triplicate. MIC was determined as the lowest concentration at which no visible growth was observed.

#### 4.4.4. Determination of Minimum Bactericidal Concentration (MBC)

MBC values were determined by selecting test tubes that showed no growth during the MIC assessment. A loop of each of these test tubes was then subcultured in Mueller-Hinton agar plates, which were incubated at 37 °C for 24 h. The experiments were conducted in triplicate. The lowest concentration of the essential oil at which no growth was observed on the agar surface was considered the MBC [162,163].

### 4.5. VirucidalActivity

#### 4.5.1. Cell Lines and Media

The human lung cancer cell line A549 (ATCC no. CCL-185^TM^), passage number 35 and the human cervical cancer cell line HeLa (ATCC no. CCL-2^TM^), passage number 30 (both lines from American Type Culture Collection, Rockville, MD, USA) were used to evaluate the virucidal activity of the preparations tested. Both cell lines were cultured in Dulbecco’s Modified Eagle Medium (DMEM) (Lonza, Basel, Switzerland), supplemented with 10% fetal bovine serum (FBS) and 4 mM L-glutamine (Biological Industries, Kibbutz Beit-Haemek, Israel). To prevent bacterial contamination during cell growth and experimentation, the medium was also supplemented with antibiotics: 100 U/mL penicillin and 100 µg/mL streptomycin (Sigma-Aldrich, Munich, Germany). These optimized conditions ensured the proper maintenance of cell cultures and enabled accurate evaluation of the virucidal effects of the substances.

#### 4.5.2. Virucidal Testing

Virucidal testing was conducted following the EN 14476 standard [161], which outlines a quantitative suspension test to evaluate the virucidal activity of disinfectants and antiseptics in medical settings. This method, established by the European Committee for Standardization (Brussels, Belgium, 2013), assesses the effectiveness of products in inactivating viruses.

To evaluate the virucidal activity of the essential oil mixture at a concentration of 1 mg/mL, the following viruses, cell lines, and reagents were used: herpes simplex virus type 1 (HSV-1-ATCC VR-1493™), human adenovirus 5 (HAdV-5, Adenoid 75 strain, ATCC VR-5™), A549 or HeLa cell lines, and phosphate-buffered saline (PBS) as an interfering substance.

The test involved the preparation of a mixture containing 0.1 mL of viral suspension (1 × 10^12^ TCID50 of HAdV-5 or HSV-1), 0.1 mL of PBS, and 0.8 mL of the test solution at the designated concentration. After a 60 min incubation period, aliquots were collected, and serial dilutions ranging from 10^−1^ to 10^−12^ were prepared. Then, 50 µL of each dilution (in eight replicates) was added to microtiter plate wells containing a monolayer of A549 cells for HAdV-5 or HeLa cells for HSV-1. Plates were observed daily for up to four days using an inverted microscope (Olympus Corp., Hamburg, Germany; Axio Observer, Carl Zeiss MicroImaging GmbH, Jena, Germany) to detect cytopathic effects (CPE) indicative of viral replication.

Residual infectivity was determined by comparing the viral titer of the test sample with that of the control. According to the standard, a substance is classified as virucidal if it achieves a ≥4 log_10_ reduction in viral titer, equivalent to ≥99.99% inactivation within the specified exposure time. The infectious dose TCID50/mL was calculated using the Spearman and Kärber method, with the following formula:

log_10_TCID50 = x_0_ − 0.5 + Σ r/n

(where x_0_ = log_10_ of the lowest dilution with a 100% positive reaction, r = the number of positive determinations in the lowest dilution with a 100% positive reaction and all higher positive dilution steps, and n = the number of determinations for each dilution step).

Experiments were performed with three independent replicates (n = 3).

### 4.6. Antioxidant Activity

The antioxidant activity of essential oils was evaluated using a model membrane system based on soybean phosphatidylcholine, following a protocol described previously described protocol with slight modifications [36,164].

Essential oils were tested at different concentration ranges depending on their activity: compounds M1, M2 and M5 from 10 to 50 μg/mL; compound M4 10 to 70 μg/mL, and M3 from 100 to 500 μg/mL.

In this test, lipid oxidation was induced by UVC radiation from a germicidal lamp (Sankyo-Denki G15T8). Before starting the experiment, the lamp was turned on and, after it had warmed up, its radiation intensity was checked, which was approximately 3.5 mW/cm^2^. The samples were then placed under the lamp. The kinetics of the oxidation process were monitored over time by collecting samples after 15, 30, 45, and 60 min of UVC exposure. Lipid peroxidation was assessed by measuring the concentration of malondialdehyde (MDA), a byproduct of oxidative degradation, which reacts with thiobarbituric acid (TBA) to form a colored complex. The degree of lipid oxidation was quantified by measuring absorbance at 535 nm using a Cary 300 Bio spectrophotometer (Varian, Palo Alto, CA, USA), where higher absorbance values corresponded to increased lipid peroxidation. Activity was measured as the percentage inhibition of the oxidation process by the compound relative to the control sample and calculated after 1 h of exposure to the formula samples:

Inhibition % = (A_o_ − A)/A_o_ × 100%

where A_o_ is the absorbance of the control sample and A is the absorbance of the sample with the compounds.

The experiments were carried out with three independent replicates (n = 3).

### 4.7. Chemopreventive Activity

#### 4.7.1. In Vitro Cell Culture

The essential oils of the mixtures were tested in four adherent cell lines. The cells used in this study were purchased from Lonza Bioscience and the European Collection of Authenticated Cell Cultures (ECACC) and stored in the biobank of the cell culture laboratory. In this test, a normal cell line, a cell line of primary normal human dermal fibroblasts, adult (NHDF-Ad, LONZA, Catalog #: CC-2511), and three cancer cell lines: a human colon cell line (LoVo, ECACC, catalog No.87060101), a lung cancer cell line (A549, ECACC, catalog No.86012804), and a breast adenocarcinoma cell line (MCF-7, ECACC, catalog No.86012803) were used. Cell lines were maintained in DMEM, DMEM F-12 medium, and EMEM medium. All cell lines were cultured in medium supplemented with 10% fetal bovine serum, L-alanyl-glutamine, gentamicin sulfate, and fungizone at 37 °C in an incubator with a 5% CO_2_ atmosphere.

Cell lines used in the tests were selected on the following properties:

NHDF—as a non-cancerous cytotoxicity control.

A549—selected due to the frequent inhalation of essential oils during aromatherapy, making the respiratory tract epithelium a suitable site of exposure.

LoVo—selected due to the potential exposure of the gastrointestinal tract through oral consumption of essential oils or food additives.

MCF-7—a hormone-dependent breast cancer cell line, included to assess the chemopreventive potential against hormone-sensitive cancers.

For SRB assays, cells were seeded in 96-well plates at densities optimized for each line to ensure monolayer formation after 24 h: NHDF—5 × 10^3^ cells/well, A549—7 × 10^3^ cells/well, LoVo—7 × 10^3^ cells/well, MCF-7—6 × 10^3^ cells/well. These conditions guarantee reproducibility and prevent overgrowth during the 48 h incubation with the test compounds.

#### 4.7.2. Evaluation of Cell Growth and Cytostatic Activity

The effects on cell cultures were evaluated using the standard sulforhodamine B (SRB) assay, a red fluorescent dye that binds cellular proteins. This method is suitable for preliminary biological research and drug discovery. Based on the results of this assay, the cytotoxic and cytostatic effects of the tested compounds were determined. Cells were seeded in 96-well plates at an appropriate density in accordance with the recommendations for the test developed by the National Cancer Institute (NCI) to screen anticancer drugs and cultured under standard conditions until they reached the desired confluence. Essential oils were diluted in DMSO to obtain the following final concentrations: 1% (without DMSO), 0.5% (0.5% DMSO), 0.25% (0.75% DMSO), 0.1% (0.9% DMSO), and 0.05% (0.95% DMSO). The cells were exposed to the appropriate concentrations of essential oils. The cells were incubated for 48 h with the test compounds. Untreated cells were used as controls. The cells were incubated for 48 h with the test compounds. Cells not exposed to the substances were used as a control. After 48 h of compound addition, the SRB test was started according to the protocol. Culture medium was removed and 50 μL of 10% TCA (Sigma, Steinheim, Germany) was added to fix the cells. The plates were then incubated at 4 °C for 1 h. After fixation, the TCA solution was removed. The plates were then air dried. Fifty microliters of 0.4% SRB solution (Sigma-Aldrich, Steinheim, Germany) were added to each well. Plates were incubated at room temperature for 30 min to allow the dye to bind to cell proteins. After staining, the plates were washed three times with 1% acetic acid (Sigma) to remove unbound dye. The plates were air-dried to remove residual acetic acid. Then, 100 μL of 10 mMTris base-solubilizing solution (Sigma) were added to each well to dissolve the bound SRB dye. After mixing, absorbance of the dissolved dye was measured at 515 nm using a microplate spectrophotometer (MultiscanGo reader, Thermo Scientific, Waltham, MA, USA). Results were expressed as a percentage of control. A negative percentage reduction corresponds to a decrease in cell number compared with untreated controls, not a negative viability. The experiments were carried out in three independent replicates (n = 3).

### 4.8. Pharmacological Tests

#### 4.8.1. Animals

Swiss albino mice of both sexes (weighing 25–30 g) were obtained from the Pasteur Institute of Algiers (Algeria). They were housed in polypropylene cages with unrestricted access to standard pellet diet and water. The animals were kept under controlled environmental conditions at a temperature of 25 ± 2 °C with a 12 h light/dark cycle. Before the experiments, the mice were acclimatized for two weeks. All procedures were carried out according to the ethical guidelines established by the Institutional Animal Ethics Committee (AASEA authorization number 85/DGLPAG/DVA/SDA/14).

#### 4.8.2. Preparation of Test Samples

Essential oil mixtures were suspended in a solution of distilled water containing 1% Tween 80 to achieve a final concentration of 100 mg/mL for oral administration [165]. All the chemicals used in this study were sourced from Sigma-Aldrich Co. (St. Louis, MO, USA).

#### 4.8.3. Acute Oral Toxicity

The acute oral toxicity assessment was carried out according to the OECD test guideline no. 423 [166]. Mice were fasted for 16 h before the experiment and allowed free access to water. Essential oils were orally administered to different groups of mice (n = 3) at doses of 50, 100, 200, 300, 500, 1000, and 2000 mg/kg. The control group received only the vehicle solution. Behavioral and autonomic responses were closely monitored for the first 2 h post administration, followed by continuous observation of mortality over the next 48 h and up to 7 days. If no fatalities were recorded in any group, the test was repeated at higher doses (3000, 4000, 4500, and 5000 mg/kg) using a new set of animals (n = 3).

### 4.9. Statistical Analysis

Results were statistically analyzed using one-way ANOVA followed by Tukey’s HSD test using R software (version 4.5.2). Values with *p* < 0.05 were considered statistically significant.

Comparative analysis of M1–M5 mixtures was conducted using a multi-faceted analytical approach to ensure objective and relevant conclusions.

## 5. Conclusions

This study demonstrates that the five examined essential oils (M1–M5) possess distinct chemical compositions and exhibit a broad spectrum of significant biological activity. All oils showed a favorable safety profile, with no mortality observed in mice at an oral dose of 5000 mg/kg. The EOs displayed strong antibacterial effects against all six multidrug-resistant bacterial strains, frequently outperforming standard antibiotic controls, and they also exhibited notable virucidal and variable antioxidant activities.

The combined EOs generally demonstrated superior antibacterial, antiviral, and antioxidant activity compared with the individual oils. Moreover, all samples showed promising chemopreventive potential, characterized by selective cytotoxicity—minimal effects on normal fibroblasts and pronounced activity against tumor cells.

Among the tested oils, M1 emerged as the most promising multifunctional candidate, demonstrating the strongest antioxidant and broad-spectrum antibacterial effects and ranking among the top anticancer agents. Its only relative limitation was moderately lower virucidal activity compared with the best-performing oils. M4 showed the highest efficacy against adenoviruses, while M3 was most effective against HSV-1. For a balanced antiviral response, M4 and M5 were the most suitable, with M5 additionally acting as a potent all-rounder across all tested activities. Although M3 showed the weakest antibacterial and antioxidant performance, it exhibited the greatest selectivity toward normal cells, which may be advantageous for applications requiring a wide therapeutic window.

Collectively, these findings underscore the potential of these essential oils as valuable components in pharmaceutical or biomedical formulations, where they may serve as modulators, adjuvants, or precursors for developing next-generation antimicrobial and chemopreventive agents. Further in vivo and mechanistic studies are warranted to fully elucidate their therapeutic potential and optimize their application.

## Figures and Tables

**Table 1:** Compounds that were significantly present in the EOs of the mixtures with a threshold of 1% (or higher).

					M1	M2	M3	M4	M5
Peak Name	tR (min)	KI Exp. SH-5	KI Lit.	Ident.	Area (%)				
***p*-Cymene**	15.858	1024	1025	KI, MS, S	**3.336**	**3.263**			**1.422**
**Limonene**	16.32	1030	1030	KI, MS, S	**6.313**	**10.438**	**22.64**	**12.403**	1.31
Eucalyptol	16.594	1033	1032	KI, MS, S			**1.592**		
**γ-Terpinene**	19.435	1057	1060	KI, MS, S	**2.598**	2.608			1.369
Linalool	24.516	1099	1099	KI, MS, S		0.926	**2.998**	0.946	
Camphor	29.888	1142	1144	KI, MS, S			**2.163**		
Menthone	31.189	1146	1148	KI, MS, S			**4.863**	0.834	
endo-Borneol	33.139	1162	1167	KI, MS			**4.116**	0.98	
**Pulegone**	41.761	1235	1237	KI, MS, S			24.542	**4.452**	
Geranial + Cinnamylaldehyde	46.57	1272	1270/1274	KI, MS, S		32.146	1.667	**50.675**	
Unknown	48.401	1289	n.d.	n.d.	**4.031**	2.556		**1.055**	2.134
**Thymol**	49.582	1290	1291	KI, MS, S	**25.095**	**15.954**		**6.26**	**8.553**
**Carvacrol**	50.472	1298	1299	KI, MS, S	41.098	**21.933**	1.091	**8.891**	
**Eugenol**	56.905	1355	1358	KI, MS, S	**1.978**		**5.556**		**61.042**
Copaene	58.873	1373	1376	KI, MS, S		1.777	0.257	1.943	
**Caryophyllene**	64.058	1403	1419	KI, MS, S			**1.062**		**10.272**
Humulene	68.352	1440	1454	KI, MS, S					1.199
α-Curcumene	72.269	1473	1485	KI, MS, S	**2.02**		**4.375**		1.376
**Zingiberene**	74.066	1493	1495	KI, MS, S	**3.992**	0.965	**5.298**	**1.309**	**2.529**
β-Bisabolene	75.537	1506	1509	KI, MS, S	**1.638**		**3.148**		**1.444**
δ-Cadinene	76.376	1520	1523	KI, MS, S		**1.434**		**1.594**	
β-Sesquiphellandrene	77.283	1524	1527	KI, MS	**1.684**		2.961		**1.174**

tR: Retention time; KI: Kovats retention index; KI lit.: literaturte retention index; MS: mass spectrum (NIST23); S: authentic standard; n.d.: no data. Bold data indicate the major components.

**Table 2:** Antibacterial activity of essential oil mixtures (inhibition zone diameter, mm). The values in parentheses indicate the standard deviation. ETP: Ertapenem (10 µg); RD: Rifampicin (30 µg).

Microorganisms	M1	M2	M3	M4	M5	Positive Control
*E. coli*	38.32 (0.15)	29.22 (0.42)	27.24 (0.15)	33.17 (0.23)	37.12 (0.00)	ETP: 10.0 (0.80)
*P. aeruginosa*	17.45 (0.12)	16.18 (0.55)	11.32 (0.14)	15.25 (0.00)	14.14 (0.23)	ETP: 30.50 (0.20)
*E. hormaechei*	32.45 (0.43)	30.12 (0.14)	27.08 (0.26)	29.19 (0.12)	28.31 (0.25)	ETP: 30.50 (0.20)
*E. auxiensis*	28.29 (0.51)	27.34 (0.12)	25.15 (0.00)	29.25 (0.45)	30.21 (0.00)	RD: 22.50 (0.80)
*K. pneumoniae*	20.39 (0.51)	18.24 (0.00)	20.18 (0.12)	17.21 (0.00)	21.27 (0.14)	ETP: 14.5 (1.20)
*S. aureus*	40.23 (0.00)	35.12 (0.32)	38.32 (0.17)	39.36 (0.41)	39.08 (0.00)	RD: 21.0 (0.50)

Values in parentheses indicate standard deviation. ETP: Ertapenem (10 µg); RD: Rifampicin (30 µg).

**Table 3:** Minimum inhibitory and bactericidal concentrations of the EOs mixture.

Microorganisms		M1	M2	M3	M4	M5
*E. coli*	MIC (μL/mL)	0.312	0.312	0.312	0.312	0.312
	MBC (μL/mL)	0.312	0.625	0.312	0.625	0.312
	MBC/MIC	1	2	1	2	1
	Activity	bactericidal	bactericidal	bactericidal	bactericidal	bactericidal
*P. aeruginosa*	MIC (μL/mL)	0.625	0.312	1.25	0.312	0.312
	MBC (μL/mL)	0.625	0.312	10	0.625	0.312
	MBC/MIC	1	1	8	2	1
	Activity	bactericidal	bactericidal	Bacteriostatic	bactericidal	bactericidal
*E. hormaechei*	MIC (μL/mL)	0.312	0.625	1.56	0.312	0.312
	MBC (μL/mL)	0.625	0.625	1.56	0.625	0.312
	MBC/MIC	2	1	1	2	1
	Activity	bactericidal	bactericidal	bactericidal	bactericidal	bactericidal
*E. auxiensis*	MIC (μL/mL)	2.5	5	5	2.5	5
	MBC (μL/mL)	5	5	10	2.5	5
	MBC/MIC	2	1	2	1	1
	Activity	bactericidal	bactericidal	bactericidal	bactericidal	bactericidal
*K. pneumoniae*	MIC (μL/mL)	5	10	5	5	5
	MBC (μL/mL)	5	10	10	5	5
	MBC/MIC	1	1	2	1	1
	Activity	bactericidal	bactericidal	bactericidal	bactericidal	bactericidal
*S. aureus*	MIC (μL/mL)	0.312	0.625	0.312	0.312	0.312
	MBC (μL/mL)	0.312	0.625	0.625	0.312	0.625
	MBC/MIC	1	1	2	1	2
	Activity	bactericidal	bactericidal	bactericidal	bactericidal	bactericidal

Mixture (M); Minimum Inhibitory Concentration (MIC); Minimum Bactericidal Concentration (MBC).

**Table 4:** Virucidal properties of EOs of the tested mixtures (log reduction and percentage reduction).

Mixtures		M1	M2	M3	M4	M5
HSV	Log	3	3.5	4.5	4	4
	%	99.9	99.95	>99.99	99.99	99.99
	SD	0	0.577	0.577	0	0
HAdV-5	Log	3.5	4	4	5	4
	%	99.95	99.99	99.99	>99.99	99.99
	SD	0.577	0	0	0	0

SD—standard deviations.

**Table 5:** IC_50_ values for the oxidation inducers UVC in the presence of M1, M2, M3, M4, and M5 mixtures.

Mixture	
	IC_50_ ± SD (μg/mL)
M1	14.02 ± 3.72
M2	27.75 ± 3.41
M3	281.60 ± 23.78
M4	60.11 ± 1.16
M5	25.90 ± 0.34

**Table 6:** Geographic data of the harvest of the species studied.

Species	Region	Geographic Coordinates
*T. vulgaris*	Kharrouba (Mostaganem)	Altitude: 80 m, longitude: 0°6′16″1 E, latitude: 35°58′742 ″N.
*A. visnaga*	Béni-saf	Altitude:25 m, longitude: 1°23′1″ O, latitude: 35°18′8″ N.
*L. angustifolia*	SebaaChioukh (Tlemcen)	Altitude: 514 m, longitude: 1°21′21 O, latitude: 35°9′22″ N.
*M. pulegium*	Abdelmalek Ramdane (Mostaganem)	Altitude: 101 m, longitude: 0°13′25 E, latitude: 36°06′46″ N.
*C. sinensis*	SidiBelAbbès	Altitude: 483 m, longitude: 0°38′29″ O, latitude: 35°12′0″ N.

**Table 7:** Content of plant components (g) in M1–M5 mixtures (100 g).

	M1	M2	M3	M4	M5
LTv	24.85	24.85			
LAv	18	18			29.6
LMp			12.74	13.16	
LLa			12.74	13.16	22.11
RZo			24.12		41.84
BCv		3.94		21.66	
CSa	3.94				6.45
PCs	53.21	53.21	50.4	52.02	
Weight	100	100	100	100	100

**Table 8:** Content of hydrodistilled essential oils (g) of mixtures (100 g), taking into account the quantities obtained from the individual components of these mixtures.

	M1	M2	M3	M4	M5
LTv	0.63	0.46			
LAv	0.45	0.33			0.95
LMp			0.34	0.28	
LLa			0.33	0.28	0.70
RZo			0.64		1.34
BCv		0.07		0.46	
CSa	0.10				0.21
PCs	1.34	0.97	1.34	1.11	
Weight	2.52	1.83	2.65	2.13	3.20

## Data Availability

The original contributions presented in this study are included in the article/Supplementary Materials. Further inquiries can be directed to the corresponding authors.

## Supplementary Materials

The following supporting information can be downloaded at: https://www.mdpi.com/article/10.3390/molecules30234579/s1. Table S1. Chemical Composition of the EOs of the Five Mixtures. Figure S1. GC-MS Chromatogram of the EO of Mixture 1. Figure S2. GC-MS Chromatogram of the EO of Mixture 2. Figure S3. GC-MS Chromatogram of the EO of Mixture 3. Figure S4. GC-MS Chromatogram of the EO of Mixture 4. Figure S5. GC-MS chromatogram of the EO from mixture 5.

## Abbreviations

The following abbreviations are used in this manuscript:

LTv	Leaves of *Thymus vulgaris*
LAv	Leaves of *Ammi visnaga*
LMp	Leaves of *Mentha pulegium*
LLa	Leaves of *Lavandulaangustifolia*
PCs	orange peel *Citrus sinensis*
RZo	Roots *of Zingiber officinale*
BCv	Bark of *Cinnamomum verum*
CSa	Clove: *Syzygium aromaticum*
M1–M5	Mixtures
