# Supplementary material for: Chemical Composition, Biological Activity, and In VivoToxicity of Essential Oils Extracted from Mixtures of Plants and Spices
Source: Molecules. 2025 Nov 28;30(23):4579. doi: 10.3390/molecules30234579 (PMC12693639; doi:10.3390/molecules30234579)
Supplement: Supplementary file 1 [file molecules-30-04579-s001.zip › molecules-3952166-supplementary.pdf]

Supplementary materials

Chemical Composition, Biological Activity, and In Vivo Toxicity of Essential Oils Extracted from Mixtures of Plants and Spices

Fouad Bahri [1,*], Antoni Szumny [2], Adam Figiel [3,*], Youcef Bahri [1], Aleksandra Włoch [4], Barbara Bażanów [5], Aleksandra Chwirot [5], Tomasz Gębarowski [6], Paulina Bugno [6], El Mokhtar Bahri [1] and Rabia Nouria Benabdeloued [1]

[1] Laboratory of Microbiology and Plant Biology, Faculty of Nature and Life Sciences, Abdelhamid Ibn Badis University, BP 188/227, Mostaganem 27000, Algeria; youcefbahri99@gmail.com (Y.B.); mokhtar.bahri.91@gmail.com (E.M.B.); benabdelouedrabia@gmail.com (R.N.B.)
[2] Department of Food Chemistry and Biocatalysis, Wrocław University of Environmental and Life Sciences, Norwida 31, 50-375 Wroclaw, Poland; antoni.szumny@upwr.edu.pl
[3] Institute of Agricultural Engineering, Faculty of Life Sciences and Technology, Wrocław University of Environmental and Life Sciences, Chelmonskiego 37a, 51-630 Wroclaw, Poland
[4] Department of Physics and Biophysics, Faculty of Biotechnology and Food Sciences, Wrocław University of Environmental and Life Sciences, C.K. Norwida 25, 50-375 Wroclaw, Poland; aleksandra.wloch@upwr.edu.pl
[5] Department of Pathology, Wrocław University of Environmental and Life Sciences, Norwida 31, 50-375 Wroclaw, Poland; barbara.bazanow@upwr.edu.pl (B.B.); aleksandra.chwirot@upwr.edu.pl (A.C.)
[6] Department of Biostructure and Animal Physiology, Faculty of Veterinary Medicine, Wrocław University of Environmental and Life Sciences, Norwida 31, 50-375 Wroclaw, Poland; tomasz.gebarowski@upwr.edu.pl (T.G.); paulina.bugno@upwr.edu.pl (P.B.)
* Correspondence: bahrifouad13@gmail.com (F.B.); adam.figiel@upwr.edu.pl (A.F.)

Table S1. Chemical Composition of the EOs of the Five Mixtures.

						**M 1**	**M2**	**M3**	**M4**	**M5**
Nr	Peak Name	tR (min)	KI exp. SH-5	KI lit.	Ident.	Area (%)				
1		8.364	886			0.044	0.045	0.007	0.012	0.019
2	α-Pinene	8.758	935	937	KI, MS, S	0.318	0.3	0.226	0.106	0.207
3	Camphene	9.716	951	952	KI, MS, S	0.141	0.009	0.488	0.007	0.151
4	Benzaldehyde	10.606	963	962	KI, MS, S	0.014	0.3	0.017	0.234	0.007
5	Sabinene	11.188	971	974	KI, MS, S	0.044	0.058	0.037	0.01	0.016
6	β-Pinene	11.564	942	943	KI, MS, S	0.042	0.049	0.045	0.021	0.018
7	β-Myrcene	12.488	993	991	KI, MS, S	0.157	0.17	0.493	0.186	0.097
8	α-Terpinene	15.037	1015	1017	KI, MS, S	0.199	0.199	0.049	0.056	0.12
9	**p-Cymene**	15.858	1024	1025	KI, MS, S	**3.336**	**3.263**	0.401	0.888	**1.422**
10	**Limonene**	16.32	1030	1030	KI, MS, S	**6.313**	**10.438**	**22.64**	**12.403**	1.31

11	Eucalyptol	16.594	1033	1032	KI, MS, S	0.207	0.076	**1.592**	0.185	0.259
12	**γ-Terpinen**	19.435	1057	1060	KI, MS, S	**2.598**	2.608	0.151	0.73	1.369
13	*cis*-Linalool oxide	20.821	1073	1074	KI, MS	0.025	0.064	0.319	0.135	0.09
14	Terpinolene	22.463	1083	1088	KI, MS, S	0.038	0.063	0.064	0.029	0.04
15	*trans*-Linalool oxide (furanoid)	22.72	1084	1086	KI, MS	0.012	0.035	0.165	0.083	0.051
16	Linalool	24.516	1099	1099	KI, MS, S	0.657	0.926	**2.998**	0.946	0.461
17	Camphor	29.888	1142	1144	KI, MS, S	0.043	0.15	**2.163**	0.666	0.392
18	Menthone	31.189	1146	1148	KI, MS, S	0.015	0.033	**4.863**	0.834	0.001
19	Benzenepropanal	32.232	1161	1164	KI, MS, S	0.002	0.332	0.306	0.528	0.002
20	endo-Borneol	33.139	1162	1167	KI, MS	0.305	0.266	**4.116**	0.98	0.441
21	terpinen-4-ol	34.268	1177	1182	KI, MS, S	0.404	0.347	0.318	0.17	0.251
22	α-Terpineol	36.321	1184	1189	KI, MS, S	0.27	0.176	0.824	0.212	0.178
23	(Z)-Cinnamaldehyde	39.417	1213	1219	KI, MS, S	0.002	0.276	0.013	0.324	0.005
24	Thymol methyl ether	41.06	1233	1235	KI, MS, S	0.222	0.173	0.064	0.048	0.08
25	**Pulegone**	41.761	1235	1237	KI, MS, S	0.146	0.084	**24.542**	**4.452**	0
26	Carvacrol methyl ether	42.172	1241	1244	KI, MS	0.019	0.097	0.025	0.015	0.009
27	Linalyl acetate	43.883	1253	1257	KI, MS, S	0.066	0.129	0.523	0.425	0.341
28	Geranial + Cinnamylaldehyde	46.57	1272	1270/1274	KI, MS, S	0.154	**32.146**	1.667	**50.675**	0.012
29	Bornyl acetate	47.836	1288	1287	KI, MS, S	0.002	0.01	0.127	-	0.003
30	Unknown	48.401	1289	n.d.	n,d,	**4.031**	2.556	0.219	**1.055**	2.134
31	**Thymol**	49.582	1290	1291	KI, MS, S	**25.095**	**15.954**	0.552	**6.26**	**8.553**
32	**Carvacrol**	50.472	1298	1299	KI, MS, S	**41.098**	**21.933**	1.091	**8.891**	0.537
33	Piperitenone	54.51	1342	1340	KI, MS, S	0.011	0.007	1.079	0.278	0.006
34	Unknown	56.768	1350	n.d.	n,d,	0.006	0.004	0.024	0.01	0.005

35	**Eugenol**	56.905	1355	1358	KI, MS, S	**1.978**	0.382	**5.556**	0.303	**61.042**
36	Cyclosativene	57.624	1364	1368	KI, MS	0.025	0.096	0.056	0.116	0.005
37	Copaene	58.873	1373	1376	KI, MS, S	0.152	1.777	0.257	1.943	0.24
38	Geranyl acetate	60.567	1380	1382	KI, MS	0.018	0.054	0.25	0.097	0.173
39	**Caryophyllene**	64.058	1403	1419	KI, MS, S	0.61	0.361	**1.062**	0.339	**10.272**
40	Humulene	68.352	1440	1454	KI, MS, S	0.048	0.106	0.456	0.297	1.199
41	Amorpha-4.11-diene	68.934	1445	1458	KI, MS	0.25	0.024	0.443	0.026	0.121
42	γ-Muurolene	71.123	1463	1477	KI, MS	0.094	0.225	0.114	0.339	0.102
43	γ-Curcumene	71.808	1470	1482	KI, MS	0.229	0.013	0.264	0.009	0.117
44	α-Curcumene	72.269	1473	1485	KI, MS, S	**2.02**	0.037	**4.375**	0.113	1.376
45	β-Eudesmene	72.954	1482	1486	KI, MS, S	0.05	0.01	0.223	0.057	0.001
46	Valencene	73.279	1483	1492	KI, MS, S	0.244	0.018	0.447	0.053	0.183
47	γ-Amorphene	73.57	1493	n.d.	KI, MS	0.352	0.001	0.641	0.005	0.225
48	Unknown	73.809		n.d.	n.d.	0.014	0.121	0.009	0.133	0.009
49	**Zingiberene**	74.066	1493	1495	KI, MS, S	**3.992**	0.965	**5.298**	**1.309**	**2.529**
50	β-Bisabolene	75.537	1506	1509	KI, MS, S	**1.638**	0.145	**3.148**	0.22	**1.444**
51	δ-Cadinene	76.376	1520	1523	KI, MS, S	0.266	**1.434**	0.375	**1.594**	0.266
52	Calamenene	76.684	1522	1524	KI, MS	0.031	0.318	0.059	0.422	0.113
53	β-Sesquiphellandrene	77.283	1524	1527	KI, MS	**1.684**	0.007	2.961	0.042	**1.174**
54	Cubenene	77.881	1532	1532	KI, MS, S	0.017	0.478	0.029	0.181	0.016
55	Caryophyllene oxide	83.031	1580	1581	KI, MS, S	0.168	0.08	0.639	0.182	0.601
56	τ-Cadinol	90.251	1634	1640	KI, MS	0.085	0.072	1.13	0.365	0.203
Total						99.779	100	100	99.999	99.924

Authentication methods: KI – Kovats retention index, MS mass spectrum (NIST23), S-authentical standard; bold data indicates the major components

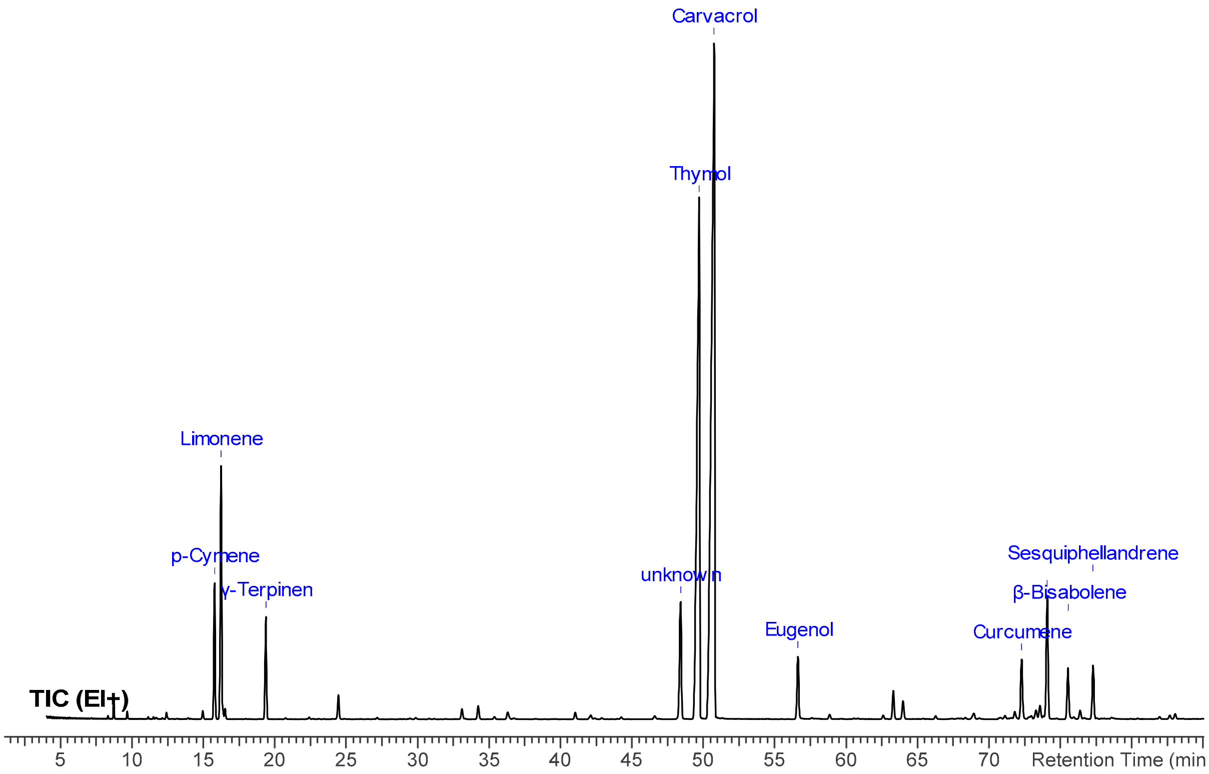

Figure S1. GC-MS Chromatogram of the EO of Mixture 1.

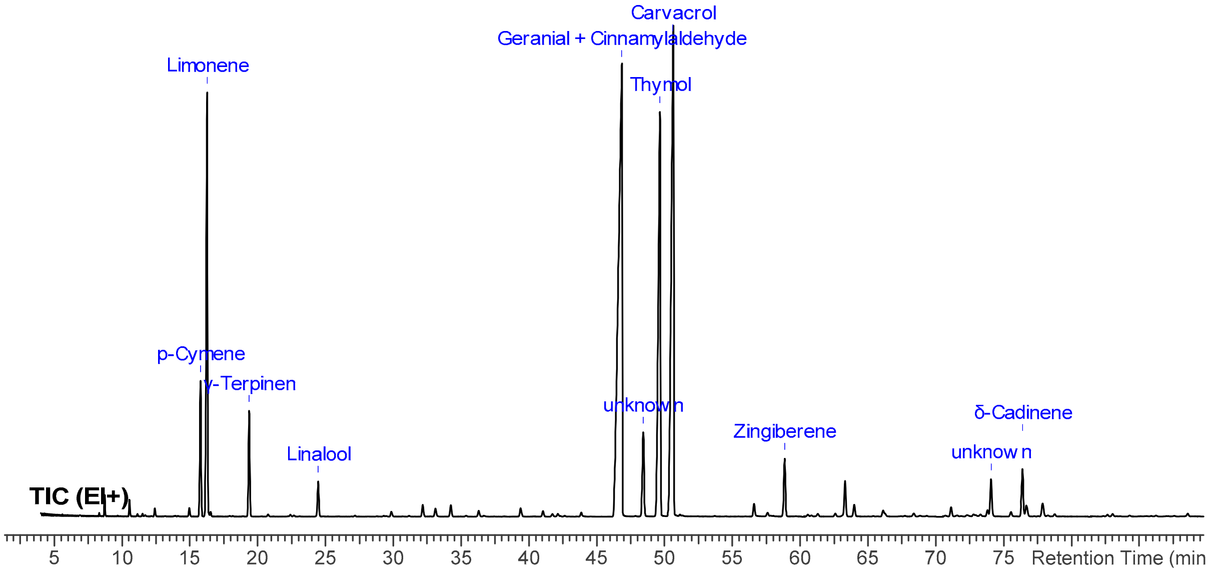

Figure S2. GC-MS Chromatogram of the EO of Mixture 2.

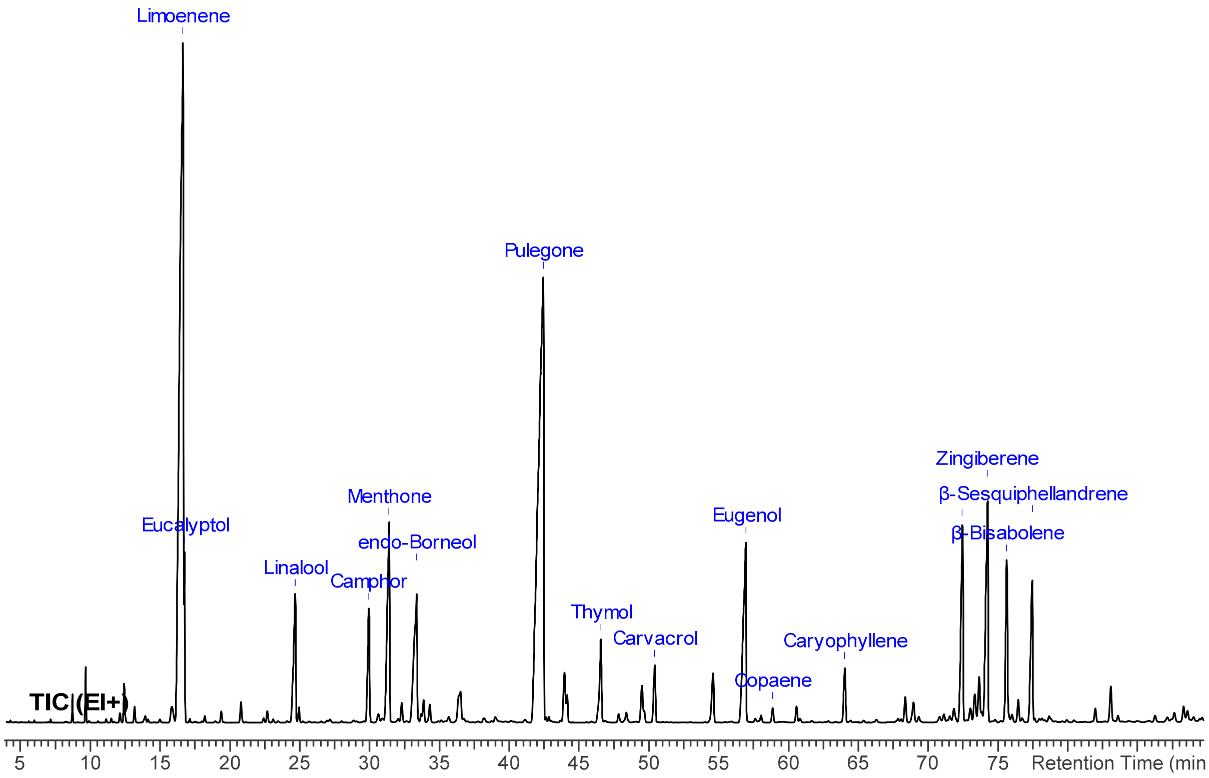

Figure S3. GC-MS Chromatogram of the EO of Mixture 3

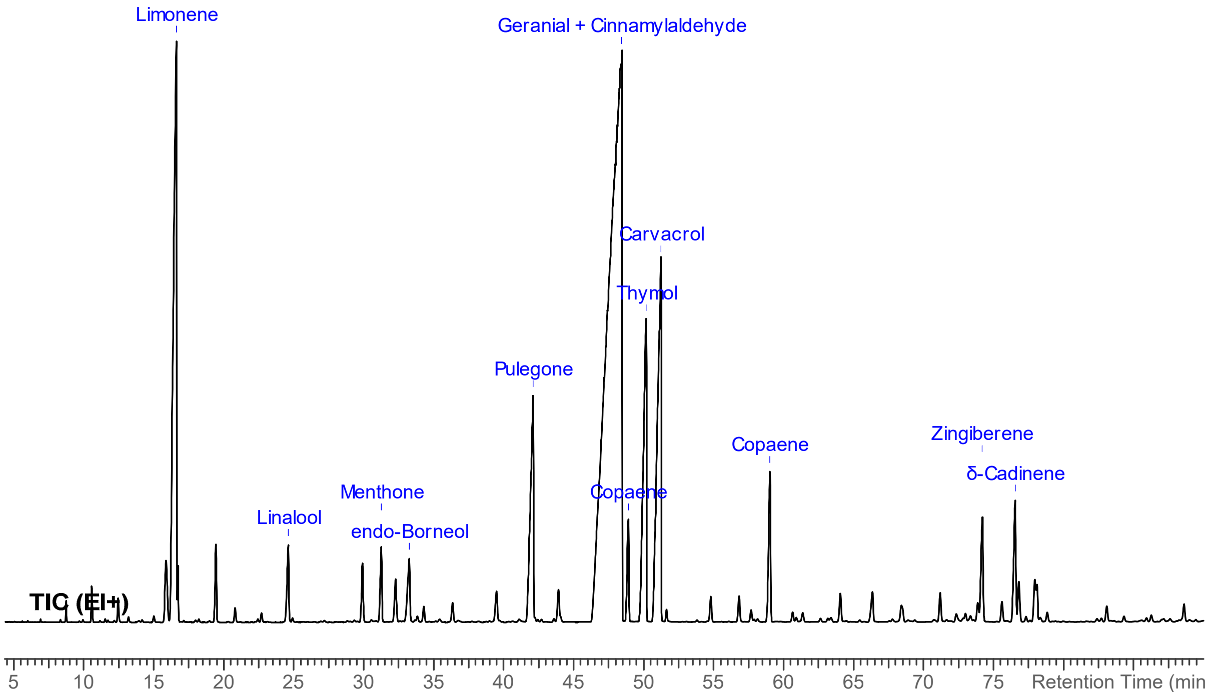

Figure S4. GC-MS Chromatogram of the EO of Mixture 4.

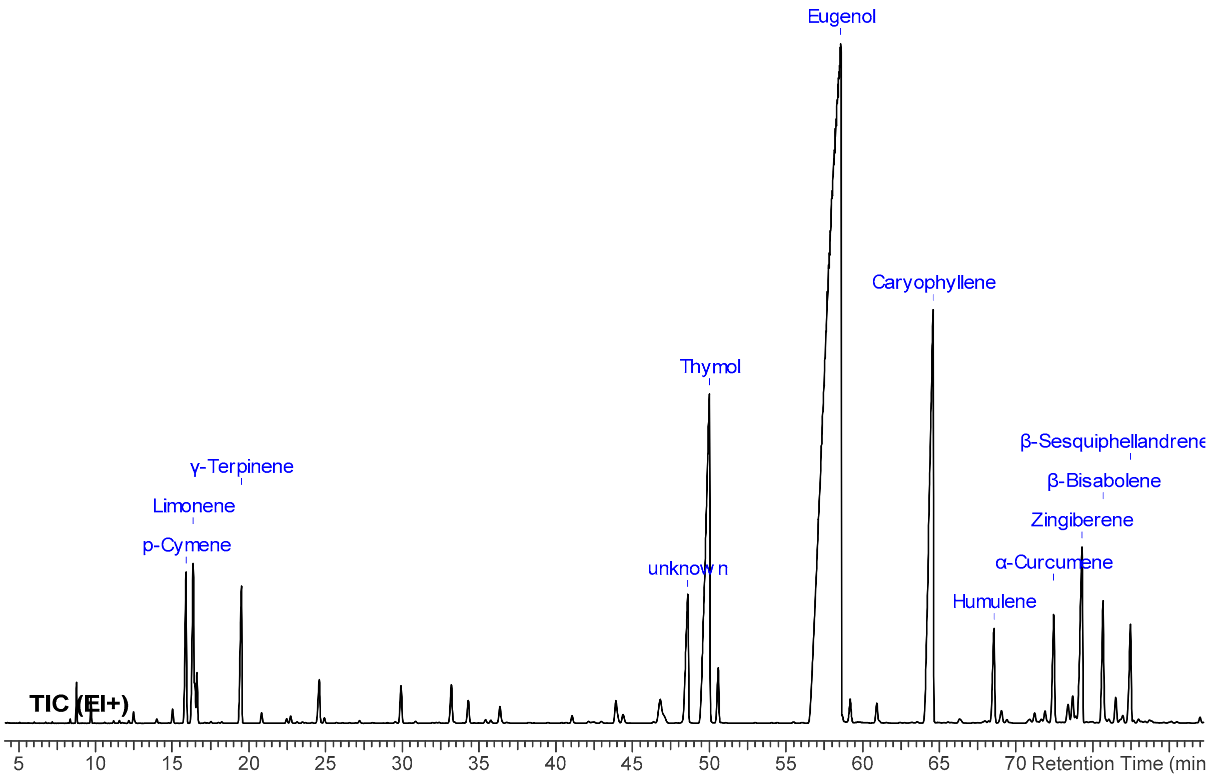

Figure S5. GC-MS chromatogram of the EO from mixture 5.
